# Dalpiciclib Combined With Pyrotinib and Letrozole in Women With HER2-Positive, Hormone Receptor-Positive Metastatic Breast Cancer (LORDSHIPS): A Phase Ib Study

**DOI:** 10.3389/fonc.2022.775081

**Published:** 2022-03-07

**Authors:** Jian Zhang, Yanchun Meng, Biyun Wang, Leiping Wang, Jun Cao, Zhonghua Tao, Ting Li, Wenqing Yao, Xichun Hu

**Affiliations:** ^1^ Department of Medical Oncology, Fudan University Shanghai Cancer Center, Shanghai, China; ^2^ Department of Oncology, Shanghai Medical College, Fudan University, Shanghai, China; ^3^ Department of Clinical Research & Development, Jiangsu Hengrui Pharmaceuticals Co., Ltd., Shanghai, China

**Keywords:** metastatic breast cancer, HER2-positive, hormone receptor-positive, pyrotinib, CDK4/6 inhibitor, endocrine therapy

## Abstract

**Purpose:**

The LORDSHIPS study aimed to explore the safety and efficacy of a novel fully oral triplet combination of dalpiciclib (a potent cyclin-dependent kinase 4/6 inhibitor), pyrotinib (a HER2 tyrosine kinase inhibitor) and endocrine therapy letrozole in patients with HER2-positive, hormone receptor (HR)-positive metastatic breast cancer (MBC) in the front-line setting.

**Patients and Methods:**

Postmenopausal women with HER2-positive, HR-positive MBC were recruited in the dose-finding phase Ib trial. A standard 3 + 3 design was used to determine safety, tolerability, and recommended phase II dose (RP2D) for the combination.

**Results:**

A total of 15 patients were enrolled to three dose combination cohorts (letrozole/pyrotinib/dalpiciclib, level/I: 2.5/400/125 mg, n=5; level/L1: 2.5/400/100 mg, n=6; level/L2: 2.5/320/125 mg, n=4). Three patients experienced dose-limiting toxicities (level/I, n=2; level/L1, n=1) and level/L2 was identified as RP2D. The most frequent grade 3-4 adverse events were neutropenia (46.7%), leukopenia (40.0%), oral mucositis (26.7%) and diarrhea (20.0%). The confirmed objective response rate (ORR) was 66.7% (95% CI: 38.4% to 88.2%). The confirmed ORR of study treatment as first line (1L) and second line (2L) HER2-targeted therapy was 85.7% (6/7) and 50.0% (4/8), respectively. Median progression-free survival (PFS) was 11.3 months (95% CI: 5.3 months to not reached). PFS in 1L setting was not reached yet, while PFS in 2L setting was 10.9 months (95% CI: 1.8 to 13.7 months).

**Conclusions:**

The fully oral combination of dalpiciclib, pyrotinib and letrozole is a promising chemotherapy-sparing treatment option for HER2-positive, HR-positive MBC patients. The planned dose-expansion phase II study is ongoing.

**Clinical Trial Registration:**

ClinicalTrials.gov, identifier NCT03772353.

## Introduction

Breast cancer (BC) is the most common cancer globally (1), with 15%-20% of BCs classified as human epidermal growth factor receptor (HER2)-positive (2). Despite successful HER2 targeted therapies, a substantial proportion of patients with HER2-positive advanced breast cancer will eventually acquire treatment resistance and succumb to their disease. The co-expression of hormone receptors (HR) is an important resistance mechanism, affecting around 50% of HER2-positive BC (2–4). Given that patients with HER2-positive, HR-positive breast cancer are less likely to respond to standard combination of anti-HER2 and chemotherapy (5–8), several studies have valuated the possibility of combined treatment with anti-HER2 and endocrine therapy. However, such regiments merely led to a modest improvement in progression-free survival (PFS) (9–11). Therefore, alternative strategies are much-needed to overcome the treatment resistance in patients with HER2-positive, HR-positive breast cancer.

Cyclin-dependent kinase 4/6 (CDK4/6) has now become a promising strategy for HER2-positive, HR-positive breast cancer treatment as it is the downstream of the estrogen receptor (ER) and HER2 pathways, as well as many other cellular pathways inducing resistance to HER2-targeted therapies (12). Preclinical studies have reported that increased levels of cyclin D1 and CDK4 confer resistance to HER2-inhibitors in tumor cells, and CDK4/6 inhibitor can regain the sensitivity to HER2-directed agents (13). Results from the MonarcHER study demonstrated that the combination of CDK4/6 inhibitor abemaciclib plus trastuzumab and fulvestrant were effective and tolerable in heavily pretreated HER2-positive, HR-positive metastatic breast cancer (MBC) patients (14). Moreover, a similar study of tucatinib, palbociclib and letrozole showed promising activity in patients with two lines of prior therapy for HER2-positive, HR-positive MBC, even in brain metastases (15, 16). Previous findings bring a glimmer of light to prevent or conquer either endocrine or anti-HER2 therapy resistance in HER2-positive, HR-positive MBC patients. However, the efficacy of the addition of CDK 4/6 inhibitors to hormonal and anti-HER2 therapies in the front-line setting remains unknown.

Dalpiciclib (SHR6390) is an oral, novel, efficient, and highly selective small-molecule CDK4/6 inhibitor (17). Phase III trial (DAWNA-1) has demonstrated improved PFS with dalpiciclib plus fulvestrant versus placebo plus fulvestrant (15.7 vs 7.2 months; hazard ratio, 0.42; p<0.0001] in pretreated HR-positive, HER2-negative advanced breast cancer (18) Pyrotinib, an irreversible pan-HER receptor tyrosine kinase inhibitor (TKI) targeting epidermal growth factor receptor/HER1, HER2, and HER4 (19), is approved for the treatment of HER2-positive metastatic breast cancer in China. In a randomized, controlled, phase III trial (PHOEBE), pyrotinib plus capecitabine yielded significantly improved PFS compared with lapatinib plus capecitabine (12.5 vs 6.8 months; hazard ratio, 0.39; one-sided p<0.0001) in HER2-positive metastatic breast cancer patients who previously received trastuzumab and taxanes (20). Notably, preclinical studies demonstrated that dalpiciclib can overcome resistance to endocrine therapy and HER2-targeted antibody in ER-positive, HER2-positive breast cancer cells (21). Additionally, dalpiciclib sensitizes pyrotinib in pyrotinib-refractory HER2-positive gastric cancer models, which has been preliminary validated in five HER2-positive gastric cancer patients (22).

Previous findings suggested that the combination of anti-HER2 agent, CDK4/6 inhibitor and endocrine therapy could be synergistic in HER2-positive, HR-positive breast cancer. To test the hypothesis, we conducted the LORDSHIPS study to investigate the safety and efficacy of a fully oral therapy that adding CDK 4/6 inhibitor dalpiciclib to the combination of pyrotinib and letrozole as front-line treatment in patients with HER2-positive, HR-positive relapsed or metastatic breast cancer.

## Patients and Methods

### Study Design and Treatments

**Figure 1:**
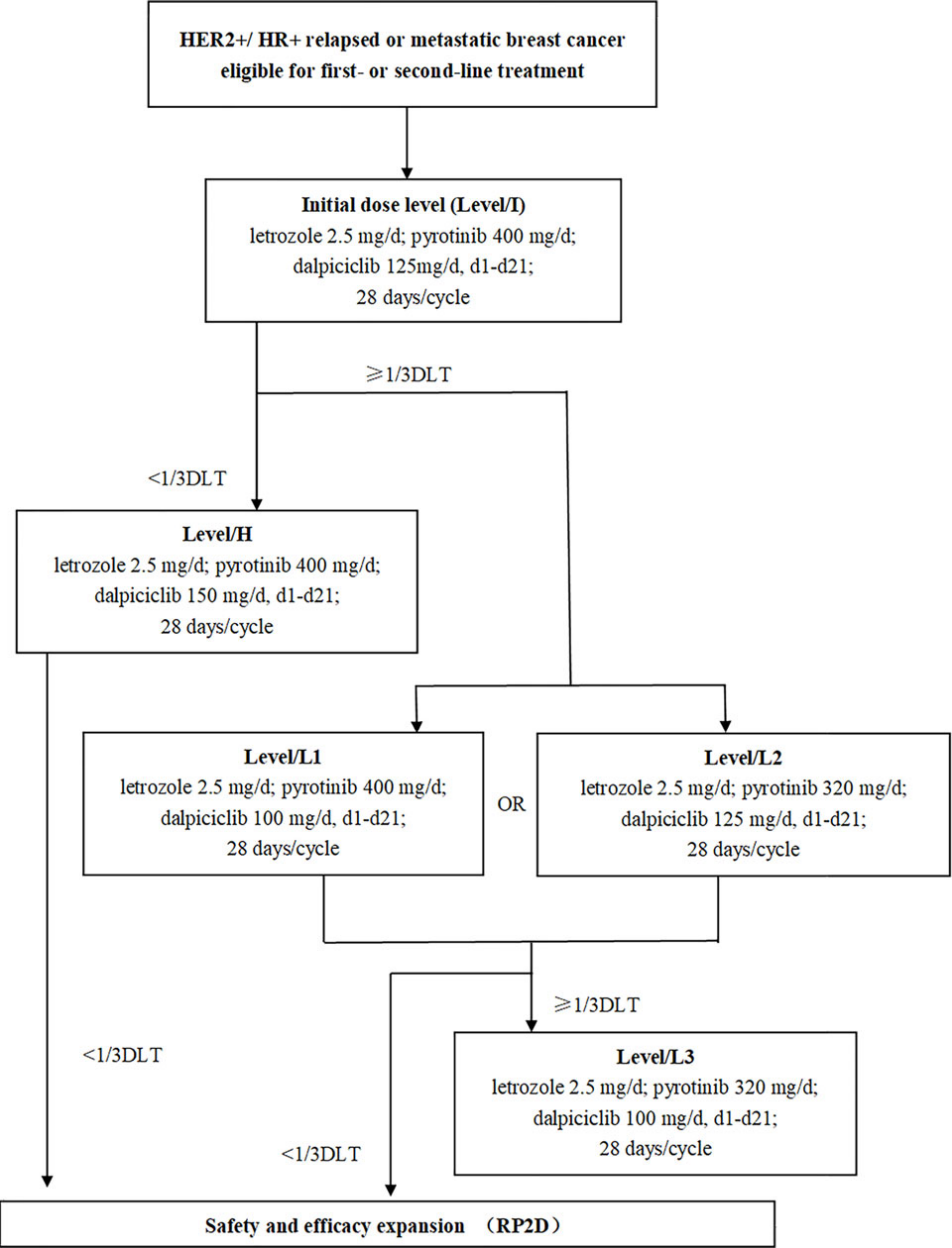
Study design. HR, hormone receptor; HER2, human epidermal growth factor receptor 2; DLT, dose-limiting toxicity; RP2D, recommended phase II dose.

The LORDSHIPS study (ClinicalTrial.gov Identifier: NCT03772353) was a single-center, open-label, dose-finding phase Ib study. In this trial, a traditional 3 + 3 design was implemented for dose finding (**Figure 1**). The treatment consisted of letrozole (fixed dose at 2.5 mg) and pyrotinib (initial dose at 400 mg) orally once daily in 28-day cycles combined with dalpiciclib (initial dose at 125 mg) orally once daily for 21 days followed by 7 days off. The combination dose finding of dalpiciclib and pyrotinib followed the “3+3” principle, with a subsequent dose escalation or de-escalation based on the incidence of specified dose-limiting toxicities (DLTs) in the initial dose group. If the initial dose level (Level/I) with pyrotinib 400 mg/d and dalpiciclib 125 mg/d could be tolerated with zero out of three patients or one out of six patients experienced a DLT, subsequent participants were assigned to the higher level (Level/H) with pyrotinib 400 mg/d and dalpiciclib 150 mg/d; otherwise, to de-escalation of dalpiciclib that Level/L1 with pyrotinib 400 mg/d, and dalpiciclib 100 mg/d, or de-escalation of pyrotinib that Level/L2 with pyrotinib 320 mg/d, and dalpiciclib 125 mg/d. If two or more patients experienced a DLT in a cohort of three or six patients at the dose of Level/L1 and Level/L2, patients would be assigned to the next dose de-escalation group in Level/L3 with pyrotinib 320 mg/d and dalpiciclib 100 mg/d according to the dose adjustment principle (**Figure 1**).

### Patients

Postmenopausal female patients aged 18-75 years, diagnosed with HER2-positive, HR-positive unresectable, relapsed or metastatic breast cancer confirmed by histopathology (local laboratory assessment) were recruited. Patients must have received ≤1 line of systemic chemotherapy for metastatic stage, ≤1 line of HER2 targeted therapy and ≤1 line of endocrine therapy. Other key inclusion criteria included at least one extracranial measurable lesion per Response Evaluation Criteria in Solid Tumors (RECIST) criteria version 1.1, an Eastern Cooperative Oncology Group (ECOG) performance status of 0-1, and adequate bone marrow and organ function. Key exclusion criteria were untreated central nervous system metastases, any prior treatment with CDK4/6 inhibitor, or proven primary resistance to letrozole or anastrozole. Primary resistance was defined as relapse during the first 2 years of adjuvant endocrine therapy or progression of disease within the first 6 months of first-line endocrine therapy for metastatic breast cancer.

The study was approved by the institutional ethics committee of the Fudan University Shanghai Cancer Center, following the principles of Declaration of Helsinki and Good Clinical Practice guidelines of the National Medical Products Administration of China. Informed consent was obtained from each participant. The potential risks and benefits of the protocol had been explained by the investigators before any study procedures were initiated. The protocol was designed and conducted in accordance with all applicable regulations, guidance, and local policies.

### Study Objectives and Assessments

The aim of the study was to determine the safety and tolerability of dalpiciclib in combination with letrozole and pyrotinib, and the recommended dose to be used in the phase II extension study.

The primary endpoints were DLTs, maximum tolerated dose (MTD), RP2D and safety of dalpiciclib in combination with letrozole and pyrotinib. DLTs were defined as the following adverse events (AEs) definitely or possibly related to study drugs in the first cycle: grade 4 neutropenia ≥5 days; grade 4 thrombocytopenia or grade 3 thrombocytopenia with significant clinical bleeding; grade ≥3 neutropenia with fever (≥38.0 degrees Celsius for 1 hour or >38.3 degrees Celsius on single oral measurement); grade ≥4 anemia; and any grade ≥3 non-hematological toxicity (excluding grade 3-4 nausea/vomiting/diarrhea/electrolyte disturbance in patients who recovered to ≤ grade 2 within 72 hours with best supportive care, and grade 3-4 increased alkaline phosphatase or glutamyl transpeptidase related to cancer instead of drugs). MTD was defined as the dose below which ≥1 of 3 or ≥2 of 6 patients experienced DLTs in the first cycle. AE severity was classified according to the National Cancer Institute Common Terminology Criteria for Adverse Events (NCI CTCAE) (version 4.0.3).

The secondary endpoints included investigator-assessed PFS, objective response rate (ORR), disease control rate (DCR), clinical benefit rate (CBR, the proportion of subjects with complete response (CR), partial response (PR) or stable disease (SD) ≥ 24 weeks during the study), and duration of response (DOR). CR and PR must be confirmed within 4-6 weeks after the criteria for response were first met. Enrolled patients underwent imaging evaluations at baseline and at the end of every 2 cycles (every 8 weeks ±7 days) until disease progression or the initiation of new anticancer therapy. The tumor response was evaluated according to RECIST 1.1 criteria. Following disease progression or initiation of new anticancer therapy, survival was followed up every 12 weeks until death. This study also collected samples for the analysis of the pharmacokinetic (PK) profile. Blood samples for PK analyses of dalpiciclib in combination with letrozole and pyrotinib were collected on day 21 of the first cycle at 1 hour, 3 hours, and 24 hours after administration.

### Statistical Analyses

All statistical analyses, except pharmacokinetic analysis (Phoenix WinNonlin 8.1), were conducted using SAS 9.2 or above (North Carolina, USA). Continuous data were presented as mean and standard deviation, or median with maximum and minimum value. Categorical data were listed as the frequency and percentage. The adverse events and serious adverse events were assessed as the indicators of safety in each dose group. Point estimates of efficacy endpoints such as ORR, DCR, and CBR were provided with 95% confidence interval (CI) calculated by Clopper-Pearson method. The Kaplan-Meier method was used to evaluate median PFS and Brookmeyer-Crowley method was used to construct 95% CI.

## Results

### Patient Characteristics

Between February 2019 and June 2020, a total of 15 eligible MBC patients from Fudan University Shanghai Cancer Center were enrolled in the phase Ib study. As of the January 1, 2022 data cutoff, the median follow-up was 11.4 months (range, 1.8-24.3 months). Four patients (26.7%) remained on study treatment, whereas 11 patients (73.3%) discontinued treatment because of disease progression (9 [60.0%]) or AEs (2 [13.3%]).

Baseline patient demographics, disease characteristics, and previous systemic therapies for breast cancer are summarized in **Table 1**. The median age was 53 years old (range, 38 to 72 years old). 14 patients (93.3%) had visceral metastases with six patients (40.0%) had more than three metastatic lesions. 10 patients (66.7%) had been previously treated with trastuzumab and 11 patients (73.3%) had received prior hormonal therapy. Seven patients (46.7%) and eight patients (53.3%) received the study treatment as first-line (1L) and second-line (2L) HER2-targeted treatment, respectively.

**Table 1:** Patient characteristics.

Characteristics	Dose Cohorts			
	Level/I (n=5)	Level/L1 (n=6)	Level/L2 (n=4)	Total (N=15)
Age, median (range), years	59 (38-65)	56 (42-72)	50 (44-55)	53 (38-72)
<65 years	4 (80.0)	5 (83.3)	4 (100.0)	13 (86.7)
≥65 years	1 (20.0)	1 (16.7)	0 (0)	2 (13.3)
ECOG performance status, n (%)				
0	0 (0)	0 (0)	0 (0)	0 (0)
1	5 (100.0)	6 (100.0)	4 (100.0)	15 (100.0)
ER status, n (%)				
ER <50%	1 (20.0)	2 (33.3)	0 (0)	3(20.0)
ER ≥50%	4 (80.0)	4 (66.7)	4 (100.0)	12(80.0)
No. of metastatic sites, n (%)				
<3	3 (60.0)	4 (66.7)	2 (50.0)	9 (60.0)
≥3	2 (40.0)	2 (33.3)	2 (50.0)	6 (40.0)
Metastatic sites, n (%)				
Visceral	4 (80.0)	6 (100.0)	4 (100.0)	14 (93.3)
Non-visceral	1 (20.0)	0 (0)	0 (0)	1 (6.7)
Previous lines of HER2-targeted treatment ^a^, n (%)				
0	1 (20.0)	4 (66.7)	2 (50.0)	7 (46.7)
1	4 (80.0)	2 (33.3)	2 (50.0)	8 (53.3)
Previous trastuzumab therapy, n (%)				
Neoadjuvant/Adjuvant only	2 (40.0)	1 (16.7)	1 (25.0)	4 (26.7)
Advanced setting	3 (60.0)	1 (16.7)	2 (50.0)	6 (40.0)
Overall	5 (100.0)	2 (33.3)	3 (75.0)	10 (66.7)
Previous endocrine therapy, n (%)				
Neoadjuvant/Adjuvant setting only	3 (60.0)	2 (33.3)	1 (25.0)	6 (40.0)
Advanced setting	2 (40.0)	1 (16.7)	2 (50.0)	5 (33.3)
Tamoxifen	2 (40.0)	3 (50.0)	3 (75.0)	8 (53.3)
Aromatase inhibitors	3 (60.0)	2 (33.3)	2 (50.0)	7 (46.7)
Overall	5 (100.0)	3 (50.0)	3 (75.0)	11(73.3)
Previous lines of chemotherapy for advanced setting, n (%)				
0	2 (40.0)	5 (83.3)	3 (75.0)	10 (66.7)
1	3 (60.0)	1 (16.7)	1 (25.0)	5 (33.3)

a 0 line anti-HER2 treatment was defined as with no history of trastuzumab treatment or relapse more than 1 year after the end of trastuzumab-based adjuvant therapy. 1 line anti-HER2 treatment was defined as relapse during or within 1 year after the end of the adjuvant trastuzumab treatment, or progression on first line trastuzumab treatment for advanced disease.

### DLTs and RP2D

Five patients were enrolled in Level/I with pyrotinib 400 mg/d, dalpiciclib 125 mg/d, and letrozole 2.5 mg/d, and two patients experienced a DLT with grade 3 oral mucositis. Subsequent participants were assigned to Level/L1 or Level/L2 with de-escalation of dalpiciclib or pyrotinib followed a 3 + 3 design. Six patients were enrolled in Level/L1 and one patient experienced a DLT with grade 3 oral mucositis, while four patients were enrolled in Level/L2 and no DLT occurred. Two different MTDs were determined as Level/L1 and Level/L2. Accordingly, Level/L2 with pyrotinib 320 mg/d, dalpiciclib 125 mg/d, and letrozole 2.5 mg/d was declared as RP2D as no DLT occurred in this cohort.

### Safety

Patients who received at least one dose of protocol therapy were evaluable for safety. All patients experienced treatment-related adverse events (TRAEs) with grade 3-4 TRAEs being reported in 80.0% of patients (**Table 2**). The most common TRAEs were neutropenia (100.0%), leukopenia (100.0%), anemia (100.0%), oral mucositis (93.3%) and diarrhea (86.7%). Other common TRAEs (≥50% of patients) included increased creatinine (73.3%), ECG T wave abnormal (60.0%) and hypertriglyceridemia (53.3%). The most frequent grade 3-4 TRAEs included neutropenia (46.7%), leukopenia (40.0%), oral mucositis (26.7%) and diarrhea (20.0%). Serious adverse events (SAE) occurred in only one patient with intracranial hemorrhage, which was attributed to cerebral arteriovenous fistula instead of study drugs. TRAEs led to dose reduction in five patients (33.3%) and treatment discontinuation in 2 patients (13.3%), respectively.

**Table 2:** All grade AEs related to treatment with at least two patients.

TRAEs, n (%)	All grades	Grade 3–4
Total patients with any AE	15 (100.0)	12 (80.0)
Hematologic		
Neutropenia	15 (100.0)	7 (46.7)
Leukopenia	15 (100.0)	6 (40.0)
Anemia	15 (100.0)	1 (6.7)
Thrombocytopenia	6 (40.0)	0 (0)
Gastrointestinal		
Oral mucositis	14 (93.3)	4 (26.7)
Diarrhea	13 (86.7)	3 (20.0)
Anorexia	4 (26.7)	0 (0)
Nausea	2 (13.3)	0 (0)
Laboratory		
Increased creatinine	11 (73.3)	0 (0)
Hypertriglyceridemia	8 (53.3)	0 (0)
Hyperglycemia	7 (46.7)	0 (0)
Hypophosphatemia	7 (46.7)	1 (6.7)
Hyperuricemia	6 (40.0)	0 (0)
Increased ALT	6 (40.0)	0 (0)
Haematuria	6 (40.0)	0 (0)
Hypokalemia	5 (33.3)	1 (6.7)
Increased AST	4 (26.7)	0 (0)
Hypoproteinemia	4 (26.7)	0 (0)
Hypomagnesemia	4 (26.7)	0 (0)
Hypocalcemia	4 (26.7)	0 (0)
Positive urine leukocyte	4 (26.7)	0 (0)
Increased ALP	3 (20.0)	0 (0)
Increased GGT	2 (13.3)	0 (0)
Hypercholesterolemia	2 (13.3)	0 (0)
Hyponatremia	2 (13.3)	0 (0)
Constitutional		
ECG T wave abnormal	9 (60.0)	0 (0)
Weight loss	7 (46.7)	0 (0)
Rash	3 (20.0)	0 (0)
Fatigue	3 (20.0)	0 (0)
Dermatitis acneiform	2 (13.3)	0 (0)
Palmar-plantar erythrodysaesthesia syndrome	2 (13.3)	0 (0)
Periodontal disease	2 (13.3)	0 (0)

ALT, alanine aminotransferase; AST, aspartate aminotransferase; GGT, γ-Glutamyl transpeptidase; ALP, alkaline phosphatase; ECG, electrocardiogram.

Note: no patients died from treatment-emergent adverse events.

### Efficacy

**Figure 2:**
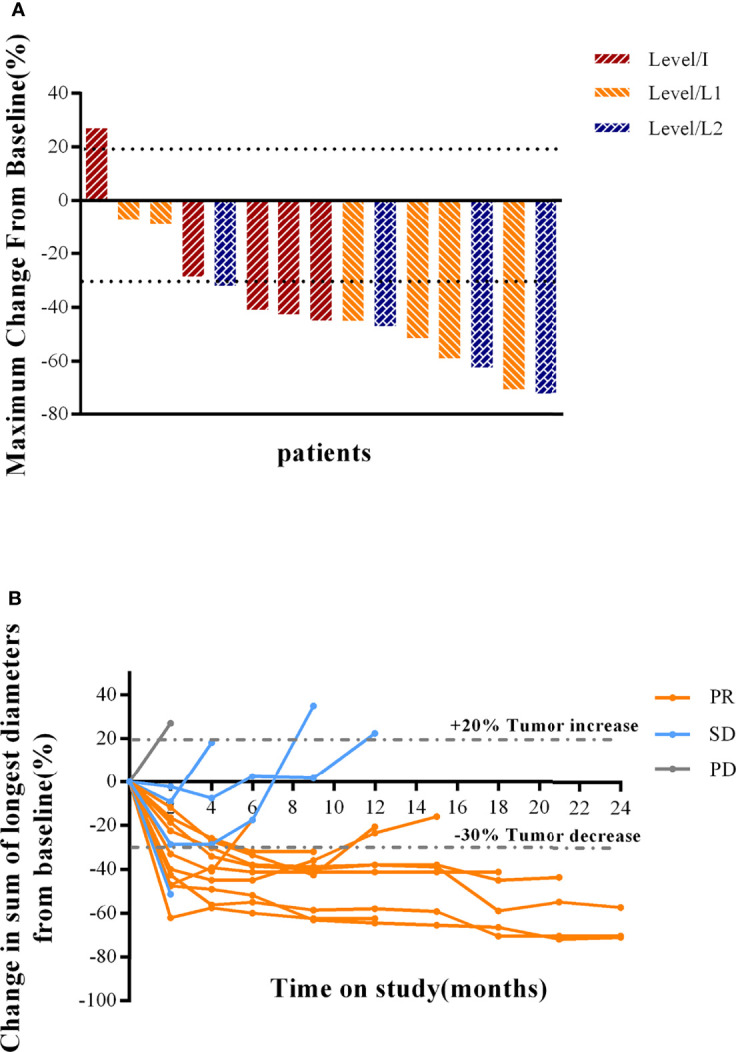
Clinical response to combination therapy in patients. **(A)** Maximum reduction of target lesions from baseline for patients in the Level/I, Level/L1, and Level/L2 dose cohorts. The best response for target lesions per patient was determined on the basis of RECIST 1.1 criteria. **(B)** Change in tumor burden over time, measured as the sum of longest diameters (SLD), in patients with MBC. PR was confirmed by investigator-assessed RECIST 1.1 criteria. PR, partial response; SD, stable disease; PD, progressive disease; HER2, human epidermal growth factor receptor 2.

**Figure 3:**
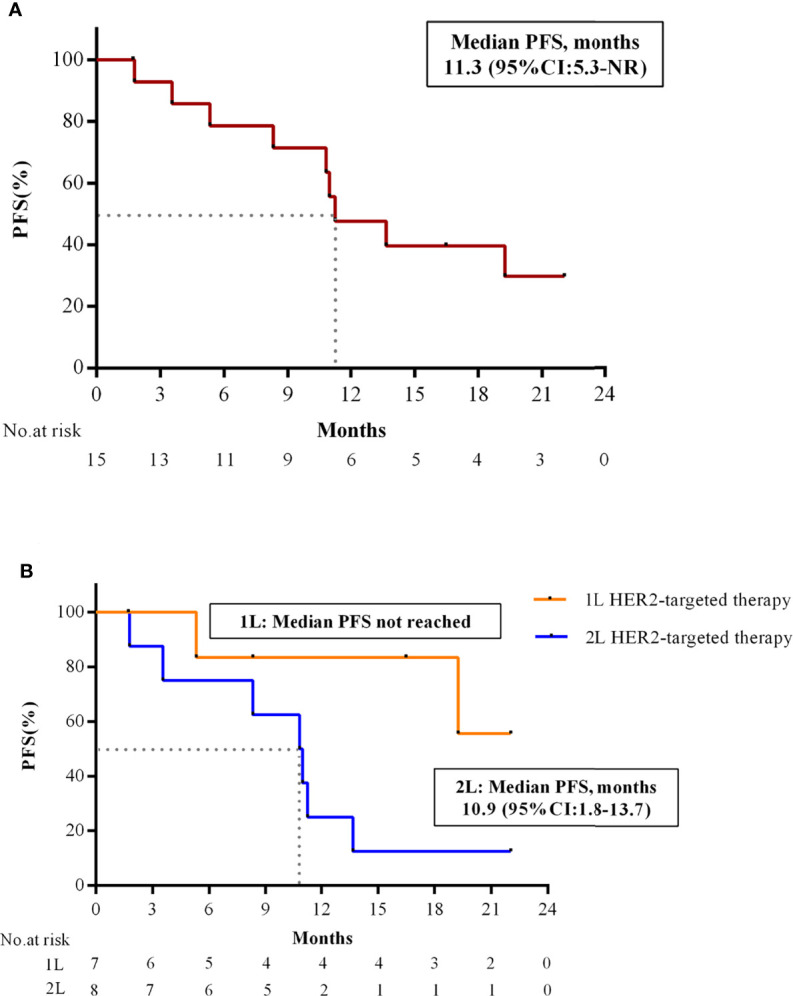
PFS of combination therapy in patients with HER2+/HR+ MBC. **(A)** Kaplan-Meier estimates of PFS in all patients (N = 15). **(B)** Kaplan-Meier estimates of PFS in patients with 1L and 2L HER2-targeted therapy. PFS, progression-free survival; HR, hormone receptor; HER2, human epidermal growth factor receptor 2; 1L, first-line; 2L, second-line; CI, confidence interval; NR, not reached.

As of 1 January 2022, 15 patients were considered evaluable for efficacy. Majority of patients (93.3%, 14/15) showed tumor shrinkage (**Figure 2A** **)**. 10 of 15 (66.7%; 95% CI: 38.4% to 88.2%) patients achieved confirmed partial response (PR) as assessed by the investigator (n=3 [60.0%], Level/I; n=3 [50.0%], Level/L1; n=4 [100.0%], Level/L2) (**Figures 2A, B** **;** **Table 3**). Responses were still ongoing in 4 of the 10 responders, and the median DOR was 15.6 months (95% CI: 3.7 months to not reached). The DCR was 93.3% (95% CI: 68.1% to 99.8%) and the CBR was 80.0% (95% CI: 51.9% to 95.7%) for all 15 patients (**Table 3**). With 9 (60.0%) disease progression events, the median PFS was 11.3 months (95% CI: 5.3 months to not reached) (**Figure 3A**).

**Table 3:** Response in the evaluable population.

Parameter	Dose Cohorts			
	Level/I (n=5)	Level/L1 (n=6)	Level/L2 (n=4)	Total (N=15)
CR, n (%)	0 (0)	0 (0)	0 (0)	0 (0)
PR, n (%)	3 (60.0)	3 (50.0)	4 (100.0)	10 (66.7)
SD, n (%)	1^a^ (20.0)	3^b^ (50.0)	0 (0)	4 (26.7)
PD, n (%)	1 (20.0)	0 (0)	0 (0)	1 (6.7)
ORR, n (%)	3 (60.0)	3 (50.0)	4 (100.0)	10 (66.7)
95% CI				38.4- 88.2
DCR, n (%)	4 (80.0)	6 (100.0)	4 (100.0)	14 (93.3)
95% CI				68.1-99.8
CBR, n (%)	4 (80.0)	4 (66.7)	4 (100.0)	12 (80.0)
95% CI				51.9-95.7

a 1 patient with SD ≥ 24 weeks.

b Among 3 patients, 1 patient with SD ≥ 24 weeks.

CR, complete response; PR, partial response; SD, stable disease; PD, progressive disease; ORR, objective response rate (CR + PR); DCR, disease control rate (CR + PR + SD); CBR, clinical benefit rate (CR + PR + SD ≥ 24 weeks); CI, confidence interval.

Preliminary subgroup analysis by the number of HER2-targeted treatment lines for advanced breast cancer showed that ORR of study treatment as 1L and 2L HER2-targeted therapy was 85.7% (6/7) and 50.0% (4/8), respectively. PFS in 2L setting was 10.9 months (95% CI: 1.8 to 13.7 months), while PFS in 1L setting was not reached yet. (**Figure 3B**). In addition, patients with ER≥50% had better ORR (83.3%, 10/12) compared with those with ER<50% (0/3).

### PK Analysis

Plasma samples for PK analysis were available from 8 patients. The PK parameters are summarized in **Supplementary 1**. The means of C_max_ for dalpiciclib (125 mg) were 130.38 ng/mL and 139.20 ng/mL, and AUC_last_ of dalpiciclib (125 mg) were 2.65 μg·h/mL and 2.52 μg·h/mL, with pyrotinib doses of 320 mg and 400 mg, respectively. Based on the preliminary data, pyrotinib did not alter the PK profile of dalpiciclib in each cohort. The exposures of pyrotinib were different when combined with dalpiciclib, indicating more data would be needed to identify the drug-drug interaction between pyrotinib and dalpiciclib in the phase II trial.

## Discussion

To the best of our knowledge, this was the first study to establish a fully oral therapy of the novel CDK4/6 inhibitor dalpiciclib combined with HER2-targeted tyrosine kinase inhibitor pyrotinib and aromatase inhibitor letrozole as first- or second-line treatment in patients with HR-positive, HER2-positive relapsed or metastatic breast cancer. This approach showed promising anticancer activities and tolerable toxicities. The TRAEs of the combination of pyrotinib, dalpiciclib, and letrozole observed in this study were as expected for each drug toxicity profile, with mild or moderate neutropenia (100%), leukopenia (100%), anemia (100%), oral mucositis (93.3%) and diarrhea (86.7%) as the most common TRAEs (17, 19, 23). Similar to dalpiciclib combined with fulvestrant in DAWNA-1 study, the incidence of hematological toxicities with dalpiciclib, pyrotinib plus letrozole was high, whereas grade≥3 neutropenia and leukopenia were reported less frequently (grade ≥3 neutropenia: 84.2% vs 46.7%; grade ≥ 3 leukopenia: 62.1% vs 33.3%) (24). Diarrhea occurred in 86.7% of patients, by only 20.0% with grade ≥ 3 diarrhea, which compared favorably with pyrotinib plus capecitabine (all grade: 95%; grade ≥ 3: 31%) (20). Diarrhea was generally reversible with anti-diarrhea treatment, treatment interruption, or dose reduction, and it did not lead to treatment termination. Three cases of oral mucositis were identified as DLTs: two cases in Level/I and one case in Level/L1 cohort, respectively. Despite the low incidence of oral mucositis with single agent [dalpiciclib, all grade: <10% and grade ≥ 3:<3% (18); pyrotinib, all grade: 9.9% and grade ≥ 3:1.4% (25)], the events were considered as possibly related to both dalpiciclib and pyrotinib. In this study, dalpiciclib 125 mg/d, pyrotinib 320 mg/d, and letrozole 2.5 mg/d was defined as the recommended phase II dose.

Regardless of the HR status, patients with HER2 overexpression/amplification should receive a combination of HER2-targeted therapy and chemotherapy as the standard 1L treatment (26). However, data from clinical trials showed that the subgroups of HER2-positive, HR-positive tumor are less likely to respond to standard chemotherapy combined with trastuzumab and pertuzumab, or with T-DM1 (5–8). In CLEOPATRA study, 1L treatment of dual HER2-targeted pertuzumab and trastuzumab plus docetaxel yielded inferior PFS and OS benefits in HR-positive/HER2-positive subsets compared to HR-negative/HER2-positive subsets (hazard ratio for PFS: 0.73 vs 0.64; hazard ratio for OS: 0.71 vs 0.61) (5). In the Chinese bridging study PUFFIN, subgroup analysis suggested that 1L treatment with dual HER2-targeted pertuzumab and trastuzumab plus docetaxel failed to prolong PFS compared to trastuzumab plus docetaxel significantly (14.5 months vs 12.5 months; hazard ratio: 0.80; 95%CI 0.50 to 1.29) in HR-positive, HER2-positive MBC patients (7). The present study showed that the median PFS in the 1L setting was not reached and the ORR was 85.7%, which was equivalent to dual-targeted HER2 agents combined with chemotherapy in CLEOPATRA (ORR in HER2-positive patients: 80.2%) (27) or PUFFIN trial (ORR in HR-positive/HER2-positive patients: 81.7%) (7). T-DM1 is the standard second-line treatment for HER2-positive metastatic breast cancer patients based on the results of EMILIA study, with an ORR of 43.6% and a median PFS of 9.6 months regardless of HR status (6). In China, pyrotinib plus capecitabine has become an alternative 2L treatment option with better PFS and OS compared to lapatinib plus capecitabine (PFS: 12.5 months vs 6.8 months; hazard ratio, 0.39; p<0.0001; ORR 67% vs 52%) (20). In our study, patients in the 2L setting had an ORR of 50.0% and a median PFS of 10.9 months (95% CI: 1.8 to 13.7 months), which was similar to standard treatment of T-DM1 or pyrotinib plus capecitabine. Although cross-trial comparisons should be made with caution, our results indicate a promising treatment option for HR-positive, HER2-positive breast cancer and support further investigations.

It’s speculated that the inferior response of anti-HER2 and chemotherapy in HR-positive subgroups compared to HR-negative subgroups was in part attributed to the bidirectional crosstalk between the HER2 and ER-α pathways (28, 29). As a result, a growing number of clinical studies have explored the combination of HER2 targeted and endocrine therapy in the subsets of breast cancer patients (9–11, 30). In TAnDEM trial (11), 1L treatment of trastuzumab plus anastrozole achieved PFS benefits compared to anastrozole (4.8 months vs 2.4 months; hazard ratio, 0.63; p=0.0016). In addition, subgroup results of phase III EGF 30008 trial showed that lapatinib plus letrozole achieved ORR 28% and PFS 8.2 months in first line patients (9). These data showed a promising but still modest PFS benefits in HR-positive, HER2-positive patients, indicating that intervention of the crosstalk between HER2 and ER-α might be insufficient and additional treatment are of value to be explored.

Preclinical models showed that CDK4/6 inhibitors could sensitize HER2-targeted therapy and delay tumor recurrence in HER2+ breast cancer (13). The monarcHER trial built on these preclinical findings and reported that CDK4/6 inhibitor abemaciclib combined with trastuzumab and fulvestrant significantly improved PFS compared to trastuzumab plus standard-of-care chemotherapy (8.3 months vs 5.7 months; hazard ratio, 0.67; p=0.051) as third-line or later treatment in HR-positive/HER2-positive MBC patients (14). Moreover, in heavily pretreated patients with HR-positive/HER2-positive MBC, the combination of tucatinib with letrozole and palbociclib showed a considerable anti-tumor activity with median PFS of 8.7 months (10.1 months for patients without brain metastasis and 6.0 months for those with brain metastasis) (15), and the central nervous system metastases PFS was 8 months in patients with untreated asymptomatic or treated stable patients with brain metastases (16). As 60.5% of HR-positive/HER2-positive MBC patients chose first-line hormonal therapy over chemotherapy in real-world (39.5%) (31), whether patients could obtain benefits from this new kind of chemo-free combination in the 1L or 2L setting would be worthy of investigation. In our study, the combination of pyrotinib, dalpiciclib, and letrozole achieving an ORR of 66.7% (95% CI: 38.4% to 88.2%) with a median PFS of 11.3 months (95% CI: 5.3 months to not reached), shows potential to be a promising chemo-sparing regimen for patients with HR-positive/HER2-positive MBC in the front-line setting. Furthermore, identifying patients who are likely to gain the most benefits from the combination of HER2-targeted and endocrine therapy with CDK4/6 inhibitor is important. Given our results, HR-positive/HER2-positive MBC patients with higher ER expression seemed to be associated with greater benefits from the combination. However, it should be noted that subgroup analysis is inconclusive due to the limited sample size.

Limitations of this early-phase study included its nonrandomized, single-arm design, small sample size and lack of direct comparator with pyrotinib plus letrozole or chemotherapy. In addition, the preliminary pharmacokinetic analyses had not yielded conclusive results because of large variation within individuals and limited blood sampling points. More patients and samples are planned to be included in further study to verify efficacy and pharmacokinetics of the combination. Meanwhile, our study excluded patients with brain metastases in phase Ib. Based on the clinical efficacy of tucatinib with letrozole and palbociclib in heavily treated patients with brain metastases, our study would further investigate the efficacy of the triplet regimen in this population in the front-line setting. Recently, T-DXd was recommended as the new standard second-line therapy by guidelines based on DESTINY-Breast03 trial with highly clinically meaningful and statistically significant improvement in PFS compared with T-DM1 in patients with HER2-positive MBC (PFS HR of 0.28 (*P* = 7.8×10^-22^)), similarly in HR-positive subgroup (22.4 months vs 6.9 months; hazard ratio: 0.3191) (32, 33). Since T-DXd has made a breakthrough in HER2-positive breast cancer, anti-HER2 ADC combined with CDK4/6 inhibitor and endocrine therapy may be the future exploring direction of HR-positive/HER2-positive breast cancer.

In conclusion, this is the first study to evaluate a fully oral treatment with the CDK4/6 inhibitor dalpiciclib plus HER2 TKI pyrotinib and letrozole in front-line HR-positive, HER2-positive MBC patients. The triplet combination of dalpiciclib, pyrotinib and letrozole has been proven to be safe and effective, potentially offering a chemotherapy-sparing treatment option for patients with HER2-positive, HR-positive MBC. The dose expansion phase II trial is ongoing to further evaluate its efficacy and safety.

## Data Availability Statement

The original contributions presented in the study are included in the article/**Supplementary Material**. Further inquiries can be directed to the corresponding author.

## Ethics Statement

The studies involving human participants were reviewed and approved by Ethics Committee of Fudan University Shanghai Cancer Center. The patients/participants provided their written informed consent to participate in this study.

## Author Contributions

All authors had full access to all data in the trial and take responsibility for the integrity of the data and the accuracy of the data analysis. JZ, YM, and XH contributed to the conceptualisation and design of the trial. BW, LW, JC, ZT, TL, and WY were responsible for collection and assembly of data. JZ and YM completed the statistical analyses. All authors participated in writing the paper and approved the final version of the paper.

## Conflict of Interest

Author WY was employed by Jiangsu Hengrui Pharmaceuticals Co., Ltd.

The remaining authors declare that the research was conducted in the absence of any commercial or financial relationships that could be construed as a potential conflict of interest.

## Publisher’s Note

All claims expressed in this article are solely those of the authors and do not necessarily represent those of their affiliated organizations, or those of the publisher, the editors and the reviewers. Any product that may be evaluated in this article, or claim that may be made by its manufacturer, is not guaranteed or endorsed by the publisher.
