# Supplementary material for: Dalpiciclib Combined With Pyrotinib and Letrozole in Women With HER2-Positive, Hormone Receptor-Positive Metastatic Breast Cancer (LORDSHIPS): A Phase Ib Study
Source: Front Oncol. 2022 Mar 7;12:775081. doi: 10.3389/fonc.2022.775081 (PMC8936075; doi:10.3389/fonc.2022.775081)
Supplement: Supplementary file 1 [file Table_1.docx]

**Supplementary 1** PK parameters of pyrotinib and dalpiciclib in Chinese patients with HER2+/HR+ MBC.

| **Drug and Doses** |  | **C_max_ (ng/mL)** | **AUClast (μg·h/mL)** | **C_min_ (ng/mL)** |
| --- | --- | --- | --- | --- |
|  |  | Level/I (n =3) | | |
| pyrotinib 400mg | mean | 143.67 | 2.78 | 86.8 |
|  | SD | 38.28 | 0.64 | 15.87 |
| dalpiciclib 125mg | mean | 139.2 | 2.52 | 75.2 |
|  | SD | 65.72 | 1.03 | 24.06 |
|  |  | Level/L2 (n =4) | | |
| pyrotinib 320mg | mean | 54.33 | 1.18 | 39.53 |
|  | SD | 14.73 | 0.34 | 14.45 |
| dalpiciclib 125mg | mean | 130.38 | 2.65 | 81.43 |
|  | SD | 44.91 | 0.84 | 21.16 |
|  |  | Level/L1 (n =1) | | |
| pyrotinib 400mg |  | 75.6 | 1.73 | 69.5 |
| dalpiciclib 100mg |  | 69.2 | 1.43 | 49.8 |

Abbreviations: PK, pharmacokinetic; HR, hormone receptor; HER2, human epidermal growth factor receptor 2; MBC, metastatic breast cancer; AUC_last_, area under the curve from the time of dosing to the time of the last measurable (positive) concentration; C_max_, peak concentration; C_min_, minimum observed concentration occurring at time T_min_; SD, standard deviation.
